# The Role of Stabilization Exercise in Preventing Pain and Postural Defects in Young Football Players

**DOI:** 10.3390/healthcare13161983

**Published:** 2025-08-12

**Authors:** Sebastian Kluczyński, Kornelia Korzan, Piotr Sorek, Tomasz Jurys, Andrzej Knapik, Anna Brzęk

**Affiliations:** 1 Department of Physiotherapy, Faculty of Health Sciences in Katowice, Medical University of Silesia in Katowice, 40-055 Katowice, Poland; korneliakorzan8@gmail.com (K.K.); piotr.sorek@interia.pl (P.S.); abrzek@sum.edu.pl (A.B.); 2 Department and Clinic of Rehabilitation, Faculty of Health Sciences, Medical University of Silesia in Katowice, 40-055 Katowice, Poland; tomasz.jurys@sum.edu.pl; 3 Department of Adapted Physical Activity and Sport, Faculty of Health Sciences in Katowice, Medical University of Silesia in Katowice, 40-055 Katowice, Poland; aknapik@sum.edu.pl

**Keywords:** youth football, core stabilization, postural control, physioprophylaxis, musculoskeletal pain, balance

## Abstract

**Background/Objectives:** Maintaining proper posture and preventing musculoskeletal pain are essential for the healthy development of young football players. Contemporary concepts of postural control emphasize the importance of the lumbopelvic-hip complex and the activation of deep trunk muscles. This study aimed to evaluate the effects of a structured core stabilization training program on postural parameters and pain reduction in young football players. **Methods**: A total of 182 male football players, aged 9–15 years, were enrolled and allocated to either the intervention or control group. The 12-week intervention consisted of exercises targeting both local and global trunk stabilizers. Assessments included measurements of spinal curvatures, trunk rotation angles, lower limb loading symmetry, and postural stability using the TMX-127 digital inclinometer (Saunders Group Inc., Chaska, MN, USA) and the Baseline scoliometer (Fabrication Enterprises, Inc. New York, USA). Pain intensity was measured using the Visual Analogue Scale (VAS). Repeated-measures statistical analyses were performed with a significance level set at *p* ≤ 0.05. **Results:** The intervention group showed significant improvements in trunk rotational parameters, with reductions in ATR values at C7/Th1 (−0.54°) and L5/S1 (−0.49°). SATR values decreased by −0.28° between the second and third assessments. Symmetry of lower limb loading under eyes-open conditions improved significantly (*p* < 0.00195). No significant changes were observed in dynamic balance, as assessed by the Y-Balance Test (*p* > 0.05). Pain intensity decreased from 3.33 to 2.55 on the VAS, reflecting a reduction of 0.78 points. **Conclusions:** Systematic core stabilization training enhances postural quality and reduces the occurrence and severity of musculoskeletal pain in young football players, with lasting effects—except for postural control under conditions of reduced visual input. This type of training represents an effective physioprophylactic strategy, supporting postural control and lowering the risk of injuries. To maintain these benefits, continued training that incorporates balance and proprioceptive exercises is recommended.

## 1. Introduction

Motor development in children and adolescents engaged in football training requires a comprehensive approach that addresses not only the enhancement of technical and conditioning skills but also the implementation of appropriate health prevention strategies [1]. A key component of contemporary physical training is stabilization exercise, which aims to improve neuromuscular coordination and activate muscles responsible for both local joint stabilization and the control of global biokinetic chains [2,3]. Particular emphasis is placed on the lumbopelvic–hip complex (LPHC), which plays a crucial role in maintaining proper posture and optimizing movement efficiency [4].

Among young football players, commonly reported musculoskeletal disorders include non-specific low back pain, pelvic asymmetry, trunk rotation abnormalities, and overload-related knee or ankle pain [5,6]. Epidemiological data indicate that up to 50–60% of adolescent footballers report recurrent spinal discomfort during periods of rapid growth, with postural misalignments identified as a major contributing factor [7]. Despite their prevalence, these issues are often underdiagnosed in youth sports contexts, potentially affecting performance, increasing the risk of injury, and predisposing athletes to chronic musculoskeletal complaints later in life.

The contemporary lifestyle of children and adolescents—characterized by sedentary behavior and reduced spontaneous physical activity outside of structured sports training—further contributes to postural dysfunction and musculoskeletal pain symptoms [8,9]. Regular incorporation of stabilization exercises into football training programs can effectively mitigate these problems by enhancing postural control and fostering motor habits that reduce the risk of overload and injury [10].

An additional challenge arises during the critical period of biological development between the ages of 11 and 13, which often coincides with intensified training loads. This phase is associated with increased vulnerability to postural abnormalities and compensatory movement patterns [11,12]. Recent studies have highlighted the high prevalence of spinal pain and postural defects during adolescence, particularly during phases of accelerated growth and increased athletic demands [10,11,12]. Forward head posture, thoracic hyperkyphosis, and lumbar hyperlordosis are frequently observed during puberty and may be exacerbated in young athletes due to sport-specific asymmetries and repetitive loading [13,14].

The implementation of a targeted core stabilization program may serve as an effective physioprophylactic strategy, contributing to both the prevention of pain and the promotion of proper postural alignment.

The aim of this study is to evaluate the role of stabilization exercises in preventing pain and postural defects in young football players. Specifically, the analysis focuses on the impact of a targeted training program on postural parameters, lower limb load symmetry, postural control, and the reduction in subjectively perceived pain.

## 2. Materials and Methods

### 2.1. Bioethics Committee

The study was approved by the Bioethics Committee of the Medical University of Silesia in Katowice (Resolution No. PCN/CBN/0022/KB1/70/21, dated 13 July 2021). All participants and their parents or legal guardians were informed about the study’s objectives, procedures, and confidentiality protocols. Written informed consent for voluntary participation was obtained from each participant’s legal guardian, with assurance of the right to withdraw from the study at any time without penalty. The physiotherapeutic intervention—a proprietary core stabilization training program—was implemented in accordance with the study protocol and followed a predetermined sequence.

### 2.2. Study Participants

A total of 182 participants aged 9 to 15 years (mean age: 11.40 ± 1.63 years), all actively engaged in football training, were enrolled in the study (Table 1). The research was conducted across three football clubs located in the Silesian and Greater Poland Voivodeships. Participants in the intervention group, who received additional core stabilization training, were randomly selected from these clubs. The control group consisted of 66 players who continued with their regular training regimen.

### 2.3. Inclusion and Exclusion Criteria for the Study

Inclusion criteria for the study comprised obtaining informed consent from the participant’s parent or legal guardian, the participant’s assent to take part in the research project, an age range of 9 to 15 years, participation in two football training sessions per week, a minimum of one year of training experience, normal body weight according to the OLA and OLAF percentile charts for age and sex, complete completion of the questionnaire form, and the absence of comorbidities [15]. Exclusion criteria included the lack of consent from the participant’s parent or legal guardian, the participant’s refusal to participate in the project, age outside the specified inclusion range, underweight, overweight, or obesity, the presence of comorbidities, incomplete completion of the questionnaire form, and the absence of systematically performed core stabilization training within the intervention group, including functional training with exercises appropriate for the first stage. 

### 2.4. Procedure for Stages I, II, and III in the Intervention Group

Following approval from the Bioethics Committee to conduct research involving young football players, a training session was held for the head coach to familiarize him with physioprophylactic strategies and to explain the rationale and structure of the functional training program. The coach was provided with a physical activity log to record player attendance, allowing for verification of adherence to the prescribed exercises. Data collection at each stage of the study was conducted with the consent of the participating clubs.

In compliance with RODO (General Data Protection Regulation) guidelines, each participant was assigned a unique identification number to ensure consistent and anonymous data tracking throughout the study. Prior to enrollment, parents or legal guardians and the participants themselves received detailed information regarding study procedures and the purpose of implementing core stabilization training. Participation required written informed consent from the legal guardian and the completion of a baseline questionnaire.

Throughout the study period, the principal investigator conducted one session of the proprietary core stabilization training per week. Training sessions were held in the sports hall and followed a predefined exercise schedule.

### 2.5. Postural Assessment

Postural alignment in the sagittal plane was assessed using the Saunders TMX-127 digital inclinometer to measure the thoracic kyphosis angle (TKA) and lumbar lordosis angle (LLA). Examinations were conducted under thermally comfortable conditions (21–26 °C) in accordance with the Saunders methodology. Anatomical landmarks were marked on the body, including C7/T1, T12/L1, and L5/S1. Participants stood barefoot in a relaxed posture with arms resting at their sides. The LLA was measured from T12/L1 to L5/S1, while the TKA was measured from T12/L1 to C7/T1. Each measurement was repeated three times, and the mean value was used for analysis [13].

Trunk rotation was assessed using a scoliometer, following SOSORT methodology. Measurements were taken at specific spinal segments, and the Sum of Trunk Rotation (SATR) was also calculated. In addition to the aforementioned landmarks, two intermediate points were marked: the apex of thoracic kyphosis (T4–T6) and the apex of lumbar lordosis (L2/L3). Participants stood barefoot with feet hip-width apart and arms flexed to 90 degrees at the shoulder joints, and then they performed the Adams forward bending test with knees extended. The examiner, standing behind the participant, placed the scoliometer at the highest point of the spine and recorded values at the designated landmarks. Each measurement was performed three times, with the mean value used for analysis.

### 2.6. Proprietary Questionnaire

To supplement data collection, a proprietary questionnaire was developed and distributed to the parents of the young football players. The questionnaire, administered in paper format, comprised 37 items, including closed-ended, semi-open, and open-ended questions. For closed-ended items, respondents selected from a five-point frequency scale (“never,” “rarely,” “sometimes,” “often,” “always”), while open-ended questions allowed for individualized responses and comments. The questionnaire design facilitated numerical coding of responses, enabling efficient statistical analysis. The items primarily addressed musculoskeletal pain complaints—both current and historical—including pain intensity, as well as the occurrence of injuries or overuse conditions.

### 2.7. Proprietary Core Stabilization Training

The training program was designed as a targeted physical activity tailored to address individual motor deficits identified during functional testing. Its primary objectives were to develop, enhance, and integrate newly acquired motor skills into daily activities and football-specific movements. This integration requires the coordinated engagement of both local and global muscle systems. Core stabilization training was structured progressively, beginning with simple, isolated tasks targeting local stabilizers and advancing to complex exercises involving global muscle activation. Moreover, benefits of core stabilization have been studied, from improving athletic performance and preventing injuries [16].

Participants in the experimental group were divided into subgroups of 15–16 players and performed exercises in pairs or groups of three. Grouping was based on similar motor deficits, enabling peer corrective feedback while minimizing unnecessary competition. Prior to introducing each new exercise, the investigator demonstrated the proper technique and provided ongoing correction during execution.

Each core stabilization session lasted from 1 to 1.5 h, depending on age group, and was conducted once weekly in a sports hall throughout the study period. In addition to these dedicated sessions, participants performed the same stabilization exercises during their regular football training twice weekly under coach supervision. The initial phase emphasized activation of deep trunk stabilizers, thoracic and shoulder girdle positioning, and coordination of muscle tension with breathing patterns.

Core stabilization sessions included approximately 10–12 exercises, individually adapted to age and skill level. Selected exercises were incorporated into warm-ups during regular football training sessions. Exercise dosage consisted of three sets of 10 to 15 repetitions, adjusted for difficulty level. Progression was contingent on the participant’s ability to maintain postural control and perform tasks pain-free. Throughout the program, emphasis was placed on movement quality and neuromuscular coordination.

Weeks I–II—Exercises were performed mainly in low and semi-low positions, with the primary aim of developing proper activation patterns of deep trunk muscles, learning thoracic and shoulder girdle positioning, and coordinating breathing with muscle engagement.

Weeks III–IV—Exercise progression was introduced by shifting towards semi-upright positions, utilizing the skills acquired in the previous weeks. For deep trunk activation, the same method of tension control was maintained in side-lying, forearm support, and seated positions on a gym ball (used as a mobile base). For the shoulder girdle, exercises were also performed in side-lying and sitting positions. Special emphasis was placed on eccentric control of scapular movement, breathing control, and the use of tonic activation of deep trunk muscles.

Week V–VI—Focus shifted to integrating all previously learned trunk stabilization, breathing, and shoulder positioning elements. Participants progressed to upright positions, with a strong emphasis on pelvic alignment control (maintaining the anterior superior iliac spine line) and preserving the long axis of the lower limbs. Closed kinetic chain exercises were performed, including the bilateral squat, hip thrust, and Bulgarian split squat, with an emphasis on both concentric and eccentric activation of the gluteus maximus while maintaining trunk stability. Shoulder girdle exercises aimed to preserve the physiological scapular angle while incorporating additional movement, such as prone support push-ups at a gym ladder and push-ups on a gym ball with controlled scapular positioning.

Week VII–IX—Unstable surface training elements were introduced to simulate sudden changes in body alignment during movement on the pitch. Equipment such as BOSU balls, Dyna-Air pads, and gym mats were used. Previously learned tasks (bilateral/unilateral squats, unilateral hip thrusts) were performed with unstable support. Additional drills included rapid directional changes with sudden stops while maintaining long-axis body alignment.

Weeks X–XII—Training content from the previous cycle was continued and enhanced with plyometric exercises aimed at developing explosive muscle strength through rapid, dynamic movements, such as jumps and throws.

### 2.8. Statistical Analysis

The collected data were consolidated into a single database and analyzed using Microsoft Excel and Statistica version 13 (Polish version). Descriptive statistics included measures of central tendency and variability, such as median (Me), arithmetic mean (M), standard deviation (SD), and 95% confidence intervals for the mean. Non-parametric tests were employed to assess differences between groups: the Mann–Whitney U test was used for two-group comparisons, while the Kruskal–Wallis ANOVA was applied for comparisons involving more than two groups. The significance level was set at *p* < 0.05.

## 3. Results

### 3.1. Characteristics of Football Training

Participants enrolled in the study provided informed consent and were aged between 10 and 15 years. All had at least one year of prior football training experience and participated in football training twice weekly throughout the study. Those assigned to the experimental group were organized into subgroups of 15–16 participants, performing exercises in pairs or small groups of three during each session. Grouping was based on similar motor deficits, enabling participants to monitor and correct each other while minimizing unnecessary competition. Prior to introducing each new exercise, the investigator gave detailed explanations of the tasks and provided continuous supervision and correction to ensure proper execution. Each core stabilization training session lasted 60 to 90 min, conducted once weekly in an indoor sports facility throughout the intervention period. Additionally, participants performed similar stabilization exercises during their regular football training sessions, held twice weekly under coach supervision.

The initial phase of the training protocol emphasized developing the ability to activate deep trunk stabilizing muscles, improving alignment of the thoracic cage and shoulder girdle, and coordinating the mechanisms that maintain physiological muscle tension in harmony with breathing patterns.

### 3.2. Qualitative Analysis of Thoracic Kyphosis Angle and Lumbar Lordosis Angle

Qualitative analysis showed a similar distribution of thoracic kyphosis angle (TKA) results in Assessment 1 for both groups, with the intervention group demonstrating a higher prevalence of values within the normative range. For the lumbar lordosis angle (LLA), a notably greater proportion of measurements fell within the normative range. In the intervention group, the number of values exceeding the normative range remained stable, while in the control group, reduced values predominated (Table 2).

**Figure 1:**
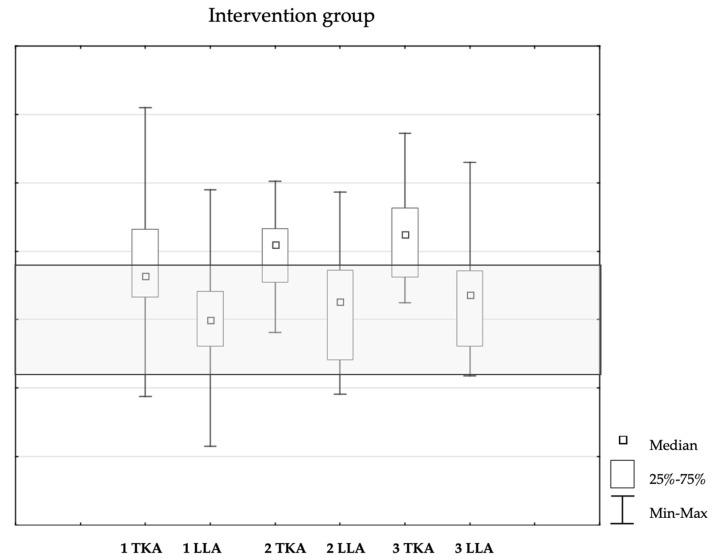
Median, Minimum, and Maximum Values of TKA and LLA Across Consecutive Assessments in the Intervention Group.

In Assessment 1, no significant differences were observed between the groups in the qualitative analysis of the thoracic kyphosis angle (χ^2^ = 1.47, df = 2, *p* = 0.48) or the lumbar lordosis angle (χ^2^ = 5.54, df = 2, *p* = 0.06). Similarly, Assessment 2 revealed no significant differences for thoracic kyphosis angle (χ^2^ = 0.49, df = 1, *p* = 0.48) or lumbar lordosis angle (χ^2^ = 5.18, df = 2, *p* = 0.07). However, analysis of three consecutive assessments of mean TKA values within the intervention group indicated a gradual increase in thoracic kyphosis angle (χ^2^ = 6.79, df = 2, *p* = 0.03), while the lumbar lordosis angle remained stable (χ^2^ = 1.29, df = 1, *p* = 0.52) (Figure 1).

### 3.3. Trunk Rotation: Sum of the Angles of Trunk Rotation (SATR)

In the entire study cohort, trunk rotation angles across the assessed spinal segments most frequently fell within the normative range, with only isolated cases reaching values between 7° and 9°. Most commonly, values of 1° were recorded in the intervention group and 3° in the control group. In both groups during Assessment 1, the mean ATR values were slightly above 3°. During Assessment 1, the mean ATR values were comparable between groups (3.26° in the intervention group vs. 3.27° in the control group), both slightly exceeding 3°. Over time, the intervention group demonstrated a subtle decrease in mean ATR between the second and third assessments (3.45° to 3.17°), while the control group exhibited an increase from 3.27° to 3.76° between the first and second assessments. Standard deviation values remained relatively stable across assessments (ranging from 1.27 to 1.49), reflecting moderate intra-group variability. Although these descriptive statistics suggest a tendency toward improved trunk symmetry in the intervention group and a potential decline in the control group. Detailed results are presented in Table 3.

**Figure 2:**
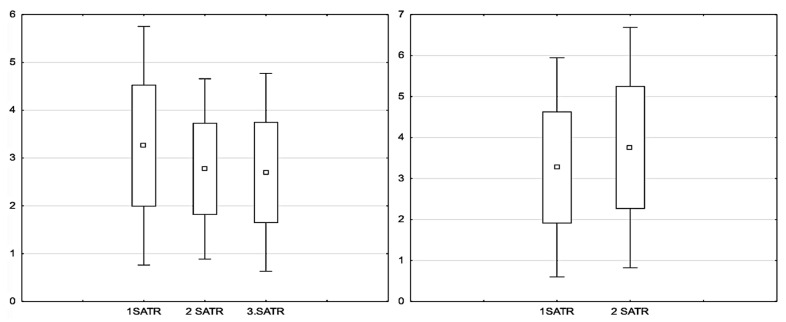
Mean, Minimum, and Maximum Values of the Sum of Trunk Rotation (SATR) on the Left: Intervention Group (**left**), Control Group (**right**).

In the intervention group, SATR values decreased across consecutive assessments, with the most pronounced reduction observed in Assessment 2 compared to Assessment 1 (Z = 3.73; *p* = 0.0002). In the subsequent assessment, the reduction was not statistically significant (Z = 1.34; *p* = 0.18). A comparison of the mean SATR values across consecutive assessments in the intervention group is presented in Figure 2 (χ^2^ = 17.84; df = 2; *p* = 0.0001).

### 3.4. Pain Complaints

**Figure 3:**
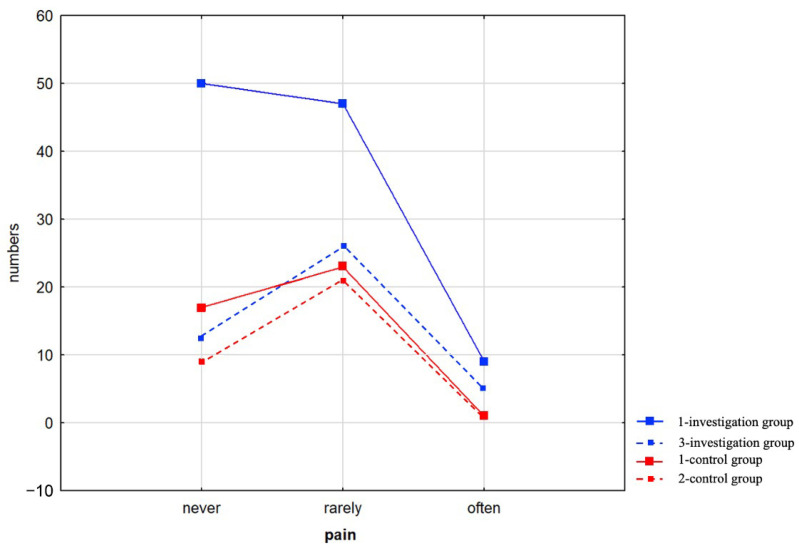
Pain Occurrence (Never, Rarely, Often) in the Assessment.

In the intervention group, 52% of participants (n = 56) reported experiencing pain, a proportion comparable to that observed in the control group (n = 24, 58%), with no statistically significant difference between the groups (χ^2^ = 2.98; df = 2; *p* = 0.224). Detailed results are presented in the figure below (Figure 3).

In the first assessment, pain intensity in both groups ranged from 1 to 9 on the VAS, with slightly higher levels noted in the control group. Following the introduction of the stabilization training program, mean pain intensity in the intervention group showed no statistically significant change between the first and second assessments (3.33 ± 2.09 vs. 3.45 ± 2.03, *p* = 0.667). Interestingly, despite stable mean values, the number of participants reporting spinal pain increased during this period. By the third assessment, however, both the prevalence of reported pain and the mean pain intensity in the intervention group decreased, with mean values reduced to 2.55 ± 1.19 and a narrower range (1–5 points). In the control group, mean pain intensity decreased slightly from the first to the second assessment (3.68 ± 2.06 to 3.26 ± 1.33), alongside a reduction in score variability (SD from 2.06 to 1.33). Detailed results on pain intensity levels are presented in Table 4.

## 4. Discussion

The findings of this study indicate that a 12-week core stabilization training program in young football players did not produce statistically significant changes in sagittal spinal curvatures. However, it contributed to improvements in trunk rotational symmetry. Within the intervention group, a slight increase in thoracic kyphosis angle (TKA) was observed, while lumbar lordosis angle (LLA) remained stable. Importantly, TKA values stayed within normative ranges, suggesting that in healthy, developing children with proper baseline posture, a 12-week intervention may be insufficient to induce structural changes in spinal alignment. It is also plausible that the slight increase in TKA reflects normal pubertal development rather than a direct effect of the training program, particularly since no growth spurts (peak height velocity, PHV) were documented during the study period.

The stability of LLA values may indicate that the regular implementation of core stabilization exercises exerts a protective effect against undesirable adaptations such as hyperlordosis, which can occur under conditions of high training load [16]. Notably, the distribution of curvature types (hypokyphosis, normokyphosis, hyperkyphosis) did not differ significantly between groups, either before or after the intervention, indicating no measurable effect of the program on static sagittal postural alignment. These observations align with prior research. For example, Sofuoğlu et al. demonstrated that an 8-week stabilization training program in young football players improved sprint performance and agility without altering spinal curvature parameters [17]. This supports the notion that in athletic youth, core stabilization exercises primarily enhance neuromuscular control and force transmission rather than directly modifying spinal structure.

Conversely, individuals with postural dysfunctions may be more likely to experience structural adaptations in response to targeted interventions. Gheitasi et al. found that adolescents with hyperkyphosis who combined corrective exercises with bracing therapy exhibited greater reductions in kyphotic angle than those using bracing alone [18]. Similarly, Gür et al. reported significant improvements in vertebral rotation and reduced back pain intensity following a 10-week stabilization training program in adolescents with idiopathic scoliosis [16].

In the present study, a significant intervention effect was observed on trunk rotation angles (ATR). The intervention group showed a consistent reduction in ATR values across all assessments, while the control group exhibited no changes. Although most participants had baseline ATR values within normal limits (~2°), improvements following the intervention suggest that core stabilization training can effectively address mild transverse plane asymmetries and enhance postural symmetry. Comparable findings have been reported in adolescents with idiopathic scoliosis, where stabilization exercises contributed to rotational correction of the spine [16]. Furthermore, a significant reduction in the sum of trunk rotation angles (SATR) was observed exclusively in the intervention group, with extreme SATR values indicative of marked postural asymmetry eliminated post-intervention. These results confirm the efficacy of stabilization training in reducing compensatory rotational asymmetries in physically active youth.

Regarding spinal pain, this study’s findings also demonstrate a positive therapeutic effect of the intervention. At baseline, over half of the participants in the intervention group reported spinal pain, consistent with literature documenting the high prevalence of back pain among young athletes. Median pain intensity on the VAS was approximately 3 points in both groups, with no significant intergroup differences initially. By the third assessment, the proportion of participants reporting spinal pain in the intervention group had decreased to roughly 50%, alongside a reduction in pain intensity. These findings suggest that systematic core stabilization exercises may contribute to long-term reductions in spinal pain among young athletes. This is supported by clinical studies such as the randomized trial by Hlaing et al., which demonstrated significant pain reduction in patients with subacute, nonspecific low back pain following core stabilization programs [19]. Similarly, Shobana et al. reported that deep trunk muscle strengthening exercises significantly alleviated chronic back pain, achieving outcomes comparable to myofascial release therapy [13]. Additionally, Liu et al. confirmed the effectiveness of a novel stabilization rehabilitation protocol in reducing chronic nonspecific low back pain [20].

In summary, core stabilization training did not significantly alter static sagittal spinal alignment in healthy young football players. Nevertheless, it positively influenced transverse plane symmetry, proprioceptive control, and neuromuscular coordination. Moreover, the intervention was associated with decreased frequency and intensity of spinal pain. These findings support incorporating structured core stabilization programs into athletic training for children and adolescents as a strategy to promote postural health, prevent injury, and optimize functional performance.

## 5. Conclusions

Systematic core stabilization training in children and adolescents engaged in football leads to a reduction in trunk rotation angles, enhancing body symmetry in the transverse plane while maintaining stability of sagittal spinal alignment. Additionally, this training reduces both the frequency and intensity of spinal pain in young football players, with beneficial effects persisting throughout the follow-up period and even after discontinuation of the program.

## 6. Limits

The primary limitations of this study stemmed from the epidemiological challenges posed by the COVID-19 pandemic, which significantly affected the intervention’s implementation. Sanitary restrictions and frequent participant absences due to quarantine and illness disrupted the continuity of the training program and limited opportunities for direct specialist supervision. Consequently, some sessions were conducted remotely or independently by participants, potentially impacting exercise quality and the consistency of training stimuli. Furthermore, restricted access to sports facilities and physiotherapy support may have diminished the overall effectiveness of the intervention. These external factors, beyond the researchers’ control, should be taken into account when interpreting the study’s results, as they may have influenced the final outcomes [21].

## Figures and Tables

**Table 1:** Variables: Age, Body Height, Body Mass, and BMI in both groups.

Group	Variable	X	SD	Me	Min	Max
**Intervention**	Age (years)	11.68	1.63	11.00	9.00	15.00
	Height (cm)	151.14	12.82	149.50	128.00	191.00
	Weight (kg)	43.14	13.19	39.15	24.80	87.50
	BMI (kg/m^2^)	18.54	3.35	17.54	13.41	31.75
	BMI percentile	57.56	16.38	50.00	25.00	99.00
**Control**	Age (years)	11.10	1.04	11.00	9.00	13.00
	Height (cm)	146.65	9.45	147.00	128.00	179.00
	Weight (kg)	38.50	7.61	37.65	26.80	63.70
	BMI (kg/m^2^)	17.75	1.96	17.44	13.75	24.54
	BMI percentile	56.56	12.91	50.00	25.00	85.00

Abbreviations: X—Mean; SD—Standard deviation; Me—Median; Min—Minimum values; Max—Maximum values.

**Table 2:** Distribution of measured parameters for thoracic kyphosis angle (TKA) and lumbar lordosis angle (LLA) across consecutive assessments in both groups (qualitative analysis).

Group	Variable	Assessment 1	Assessment 2	Assessment 3
		N (%)	N (%)	N (%)
**Intervention Group**	TKA (°)—Increased	52 (44.83%)	29 (65.91%)	21 (70%)
	TKA (°)—Normative	62 (53.45%)	15 (34.09%)	9 (30%)
	TKA (°)—Reduced	2 (1.72%)	-	-
	LLA (°)—Increased	13 (11.21%)	11 (25.00%)	6 (20%)
	LLA (°)—Normative	90 (77.59%)	25 (56.82%)	23 (76.67%)
	LLA (°)—Reduced	13 (11.21%)	8 (18.18%)	1 (3.33%)
**Control Group**	TKA (°)—Increased	33 (50%)	24 (58.54%)	-
	TKA (°)—Normative	33 (50%)	17 (41.46%)	-
	TKA (°)—Reduced	-	-	-
	LLA (°)—Increased	5 (7.58%)	3 (7.32%)	-
	LLA (°)—Normative	45 (68.18%)	31 (75.61%)	-
	LLA (°)—Reduced	16 (24.24%)	7 (17.07%)	-

Abbreviations: N—Number; %—percentage; TKA—thoracic kyphosis angle; LLA—lumbar lordosis angle.

**Table 3:** SATR values across consecutive assessments in the intervention and control groups.

Group	Assessment	M	SD	Me	Min	Max
**Intervention**	Assessment 1 (n = 116)	3.26	1.27	1.00	1	8.00
	Assessment 2 (n = 44)	3.45	1.35	1.00	1	8.00
	Assessment 3 (n = 30)	3.17	1.34	1.00	1	7.00
**Control**	Assessment 1 (n = 66)	3.27	1.37	3	1	9.00
	Assessment 2 (n = 41)	3.76	1.49	4	1	8.00

Abbreviations: M—mean; SD—standard deviation; Me—median; Min—minimum value; Max—maximum value.

**Table 4:** Variables: Pain Intensity Values on the VAS.

Group	Assessment	M	SD	Me	Min	Max
**Intervention**	Assessment 1 (n = 116)	3.33	2.09	3.00	1.00	9.00
	Assessment 2 (n = 44)	3.45	2.03	3.00	1.00	9.00
	Assessment 3 (n = 30)	2.55	1.19	2.50	1.00	5.00
**Control**	Assessment 1 (n = 66)	3.68	2.06	4.00	1.00	9.00
	Assessment 2 (n = 41)	3.26	1.33	3.00	1.00	7.00

Abbreviations: M—mean; SD—standard deviation; Me—median; Min—minimum value; Max—maximum value.

## Data Availability

Data are contained within the article.
